# Methotrexate-Associated Lymphoproliferative Disease of the Thoracic Spine Misdiagnosed as Metastatic Spinal Tumor: A Case Report

**DOI:** 10.7759/cureus.27692

**Published:** 2022-08-04

**Authors:** Masatsugu Tsukamoto, Tadatsugu Morimoto, Hirohito Hirata, Tomohito Yoshihara, Masaaki Mawatari

**Affiliations:** 1 Department of Orthopaedic Surgery, Saga University, Saga, JPN

**Keywords:** mtx-lpd, lymphoproliferative disorder, treatment, rheumatoid arthritis, tumors, spinal lesions, lymphoma, methotrexate-associated lymphoproliferative disease, methotrexate

## Abstract

Methotrexate (MTX) is increasingly used in the treatment of rheumatoid arthritis. Many recent reports have identified MTX-related lymphoproliferative disorder (MTX-LPD) as lymphoma that develops during MTX therapy. However, spinal lesions, which are extremely rare, can be misdiagnosed as spinal metastases or pyogenic spondylitis. Here, we describe a 69-year-old man with rheumatoid arthritis who had MTX-LPD of the thoracic spine. He complained of back pain and weakness in the bilateral iliopsoas muscle. A radiographical assessment by his previous physician revealed the cause to be a spinal tumor. They performed posterior spinal decompression and fixation, and a pathological examination revealed only inflammatory changes, necrosis, and increased collagen fiber growth, with no evidence of malignancy. Nevertheless, magnetic resonance imaging two weeks after the surgery showed an increase in the size of the spinal tumor. When the lesion paralyzed the patient soon afterward, the physician considered that a total en bloc spondylectomy was necessary and referred the patient to our hospital. MTX-LPD was suspected because of a history of MTX administration, and a biopsy, posterior spinal decompression, and fixation were performed again. Following the histopathological diagnosis of the tumor as MTX-LPD, MTX administration was terminated. Three months following surgery, the tumors' removal was confirmed. Because MTX-LPD can be treated with MTX withdrawal, correct diagnoses should be made, and unnecessary treatments avoided.

## Introduction

Rheumatoid arthritis (RA) is treated with the immunosuppressive drug methotrexate (MTX), an antimetabolite. However, MTX therapy is widely known to cause serious adverse effects, including liver disorders, interstitial pneumonia, and myelosuppression, among others.

The first instance of MTX-related lymphoproliferative disorder (MTX-LPD) was described by Ellman et al. in 1991 as lymphoma developing in RA patients receiving MTX [1]. Following that, numerous reports of MTX-LPD were published, especially ones from Japan. MTX-LPD was listed in the 2008 World Health Organization histological classification of lymphoid neoplasms.

Spinal lesions associated with MTX-LPD, which are extremely rare, can be misdiagnosed as spinal metastases or pyogenic spondylitis. Here, we report a case of MTX-LPD in the thoracic spine of a patient with RA who was being treated with MTX.

## Case presentation

A 69-year-old rheumatoid arthritis (RA) patient reported bilateral iliopsoas muscle weakening (manual muscle testing (MMT) grade 3) and back pain. He had been on MTX (10 mg/week) for approximately 10 years (approximate cumulative dose of MTX: 2000 mg).

**Figure 1:**
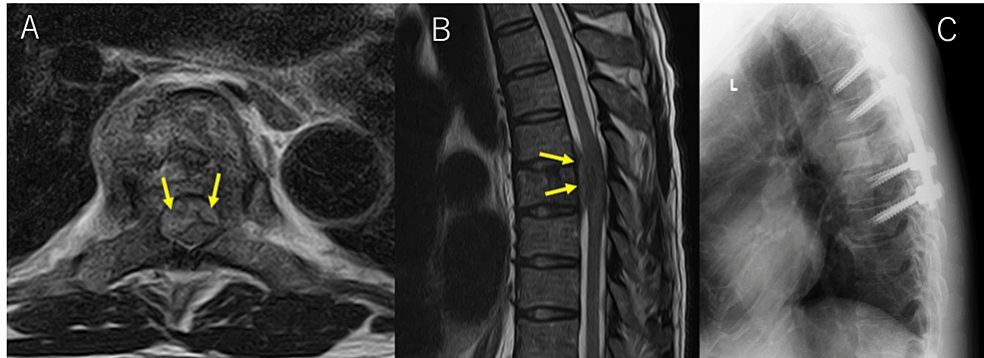
Initial preoperative MRI and postoperative radiograph of the first surgery (A, B) Axial (A) and sagittal (B) T2-weighted MR images were obtained at the Th7 vertebra at admission, showing an epidural mass-causing cord. (C) Lateral radiograph of the thoracic spine postoperatively after the first surgery.

His prior medical professional discovered a spinal tumor after a radiographic examination (Figures 1A-1B). A computed tomography (CT) scan of the entire body revealed no malignant lesions in the patient's major organs, and the patient had no history of cancer.

On admission to the previous hospital, the patient's bilateral iliopsoas weakness progressively worsened, making it impossible for him to walk. He underwent posterior spinal decompression with a laminectomy and posterolateral fixation of the Th5-9 vertebrae as a result, along with a biopsy (Figure 1C).

However, the biopsy only detected inflammatory changes, necrosis, and increased collagen fiber growth, with an absence of malignancy. Nevertheless, magnetic resonance imaging (MRI) two weeks after the surgery showed an increase in the size of the spinal tumor. His physician judged that total en bloc spondylectomy (TES) was necessary, and the patient was referred to our hospital for further treatment.

On admission to our hospital, initial testing showed bilaterally enhanced patellar and Achilles reflexes, bilaterally weak iliopsoas muscles, an MMT score of 3, and bilaterally impaired sensory function in the posterior lower legs.

Laboratory tests showed normal lactate dehydrogenase levels (134 IU/L), elevated C-reactive protein level (9.4 mg/ml), a slight elevation in the soluble interleukin-2 receptor value (1370 U/ml), and an increased Epstein-Barr virus (EBV) immunoglobulin G antibody titer. The range of tumor markers was normal.

**Figure 2:**
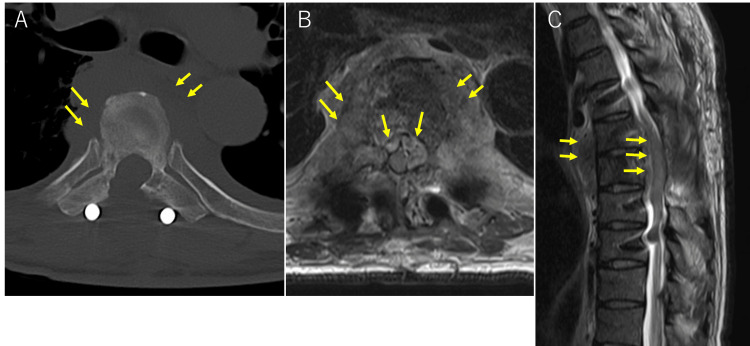
Postoperative CT and MRI after two weeks from the first surgery Axial plane CT (A), axial (B), and sagittal (C) T2-weighted MRI revealed a soft-tissue mass, which extended to the epidural space and paravertebral lesion.

A soft-tissue mass that spread to the epidural space, mediastinal region, and paravertebral lesion in the T7 vertebral body was discovered by CT and MRI scans (Figures 2A-2C).

A case of MTX-LPD was suspected based on physical examination, blood tests, and MRI findings; however, other differential diagnoses suggested spinal metastases as well as myeloma and inflammatory changes. We, therefore, planned a re-biopsy, which, due to a walking disability, was performed along with another posterior spinal decompression and posterolateral fixation of the Th5-9 vertebrae. A dark brown malignant lesion was found on the dorsal side of the dura mater at the Th7 vertebral level, according to the intraoperative results. We removed as many epidural lesions as we could without harming the dura since they were securely adherent to the dura, and we were unable to remove all of them. Pathological specimens were also collected. An intraoperative rapid pathological examination suggested the presence of lymphoma.

**Figure 3:**
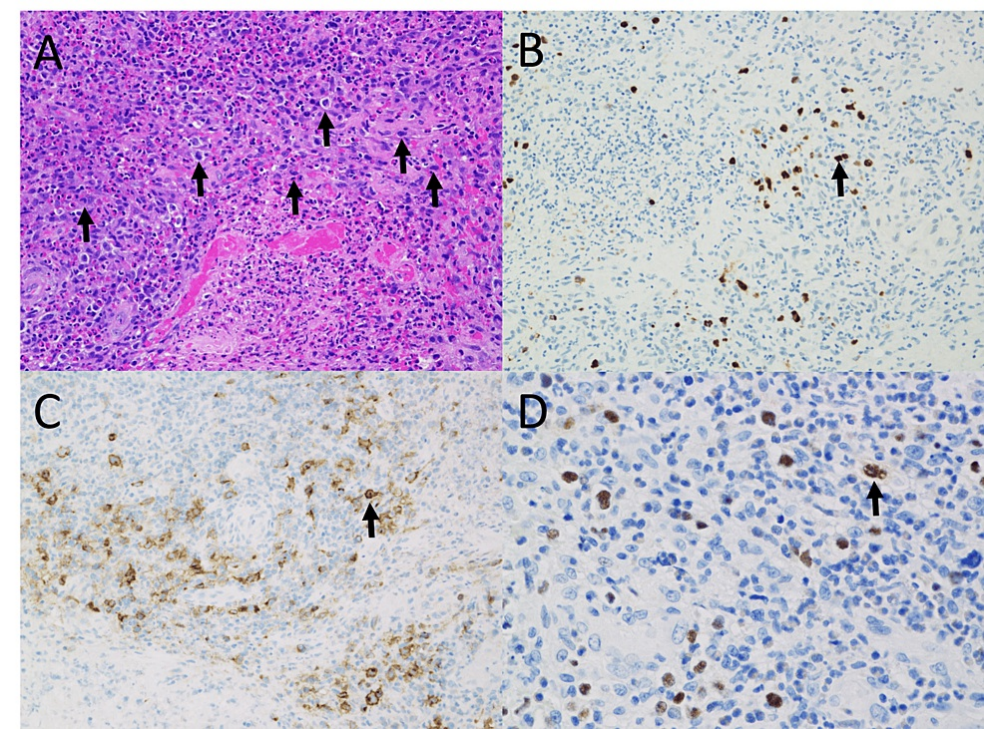
Histologic findings of the tumor in a Th8 spinal canal lesion (A) Hematoxylin-eosin stain demonstrated extensive necrotic nest formation, with a mixed collection of lymphocytes, eosinophils, and macrophages at the margins, in which large atypical cells with enlarged nuclei were observed. (B-D) Findings from the immunohistochemical analysis. The large atypical cells with enlarged nuclei were EBER-positive (B), and the positive findings for CD30 were almost consistent (C). The large atypical cells were also positive for PAX5 (D). These findings were diagnostic to support Hodgkin's lymphoma-like LPD.

Final pathological findings revealed the formation of an extensive necrotic layer, the margins of which contained areas of aggregates of lymphocytes, eosinophils, and macrophages, among which were large atypical cells. The nuclei of the large atypical cells were enlarged (Figure 3A). The Epstein-Barr encoding region in situ hybridization indicated EBV positivity. Immunohistochemical examination showed large atypical CD30-positive cells and scattered distribution of PAX5-positive cells, suggesting a diagnosis of Hodgkin’s lymphoma-like LPD (Figures 3B-3D).

**Figure 4:**
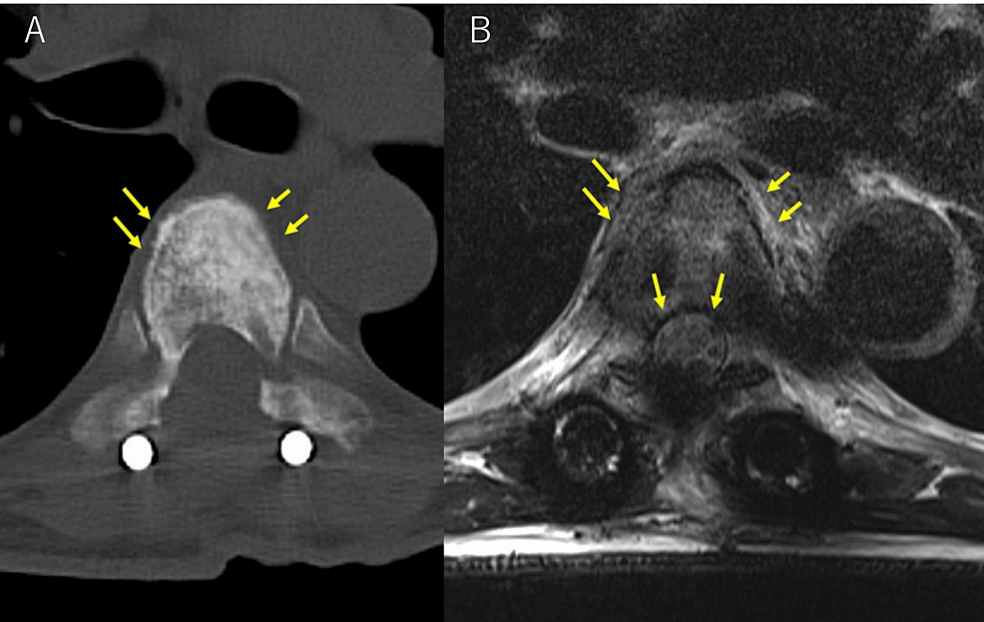
CT and MRI at three months after MTX discontinuation CT (A) and T2-weighted MRI (B) in the axial position confirmed the disappearance of a soft tissue mass extending into the epidural space and paraspinal lesions.

Considering these pathological findings, the history of MTX treatment and EBV positivity, the patient was diagnosed with MTX-LPD. His MTX therapy was discontinued based on a working diagnosis of MTX-LPD. After the discontinuation of MTX, we observed an improvement in his symptoms and shrinkage of the tumor, therefore leading to a definitive diagnosis of MTX-LPD. The patient's postoperative bladder rectal dysfunction and bilateral iliopsoas muscle weakness both showed recovery, and he was able to walk again. Soluble interleukin-2 receptor levels decreased to 565 U/ml one month after MTX discontinuation. An anti-EBV capsid antigen immunoglobulin G (IgG) level that was 5.7 times higher than normal (less than 0.5) during a postoperative test indicated a history of infection. The symptoms of RA did not worsen. A contrast-enhanced CT scan performed two weeks following MTX discontinuation showed that a residual tumor on the lateral side of the vertebral body had begun to shrink. The results of the MRI showed a decompressed spinal cord and a partially resected epidural tumor mass. Within three months of the procedure, it was determined that both tumors had vanished (Figures 4A-4B). By this time, the patient could walk by himself and there were no signs of tumor recurrence one year after the surgery.

## Discussion

Patients with suspected spinal metastases should have a thorough diagnostic work-up, including a history assessment, physical examination, and imaging studies. Although advances in imaging technology have improved the detection of cancerous lesions, tissue samples from spinal masses are often still required for definitive diagnoses. For cases of spinal tumors with advancing symptoms, a proper diagnosis is essential for determining the best course of treatment since chemo- or radio-sensitive tumors can be treated without spinal surgery, which has a complication risk of 21-26% [2-3]. Up to 6.7-10% of spinal metastases have no known source [4-5]. If surgery and excisional biopsy are not immediately indicated, a percutaneous biopsy may be required because most treatment decisions will be dictated by the histological findings of the tumor. However, in order to collect the diseased material and decompress the spinal cord in cases of tumors that quickly paralyze patients, emergency spinal surgery should be done.

The initial case of MTX-LPD very recently came to light and was based on the genesis of a malignant lymphoma during MTX treatment, which, along with the associated clinical symptoms, spontaneously remits when MTX administration is stopped. Although the exact mechanism of MTX-caused pathogenesis is unknown, the number of reports is steadily growing. This disorder has been reported more frequently in Japan than in other nations, but it is unclear if this is due to a local perception or racial differences [6].

MTX-LPD is a general phrase that refers to all LPDs that arise in patients receiving MTX therapy. It could encompass both typical LPD and LPD associated with RA, both of which are difficult to distinguish. The most prevalent extra-lymphatic lesions associated with MTX-LPD are ones in the gastrointestinal system, skin, liver, and lungs [7]. The musculoskeletal system can be affected by MTX-LPD, with bone marrow infiltration accounting for around 3% of all known MTX-LPD cases [8]. It is extremely uncommon for the bone marrow to invade the spinal column and to our knowledge, there have only been three reported cases of MTX-LPD of the spine [9-12]. Kikuchi et al. reported a rare case of MTX-LPD originating from the lumbar spine for the first time [9]. The current case is the fourth known case to the best of our knowledge.

The clinical features of MTX-LPD include a history of MTX treatment for at least 30 months, a history of EBV infection, and the potential for remission following the cessation of MTX [13]. The present case showed all characteristics of MTX-LPD: a history of MTX medication at a dose of 10 mg/week for 10 years; a high anti-EBV capsid antigen-IgG level; and complete remission within six months after MTX withdrawal. As a risk factor for LPD in RA patients, Kameda et al. found a statistically significant difference in the dose of MTX alone [8].

The most common treatment option is the discontinuation of MTX. Although no strict guidelines for the treatment of MTX-LPD have been established, the withdrawal of MTX under observation has usually been considered to be beneficial. According to previous studies, over half of the MTX-LPD cases respond to MTX withdrawal alone [7-8]. In other reports, spontaneous regression was observed in 23 (53.5%) of 43 patients with MTX-LPD who were then followed up with MTX discontinuation; EBV positivity was significantly higher in the spontaneously regressed patients (85.2%) than in the non-regressed patients (50%) [14]. On the other hand, there have been cases of rapid progression and cases of an increase after regression. After looking at published data from 26 patients, Rizzi et al. found that complete remission typically happens four weeks after stopping MTX and other immunosuppressive medications [15]. On the other hand, Inui et al. found that in 13 out of 15 patients, the greatest tumor shrinkage didn't happen until eight weeks after stopping MTX [16]. In order to assess the tumor's response, regular follow-up should proceed after eight weeks since the interruption of MTX. According to Okuwaki et al., when MTX was stopped, a case of recurrent MTX-LPD that had developed in the lumbar spine spontaneously remitted, but the patient relapsed 12 months later [12]. MTX-LPD can relapse even after remission has been achieved, suggesting the need for careful follow-up observations.

## Conclusions

Here, we report a rare case of an MTX-LPD originating in the thoracic spine. The patient, who had been receiving treatment for RA for 10 years, was initially misdiagnosed with a spinal tumor. A correct diagnosis, obtained through careful evaluation of his medical history and biopsy findings, allowed for prompt treatment in the form of simple discontinuation of MTX. Although spontaneous regression was achieved by MTX discontinuation alone, the patient will require continuous and rigorous follow-up in six-month intervals. MTX-LPD should be considered one of the differential diagnoses of spinal tumors in patients receiving MTX therapy and thus be screened properly to assure diagnosis in relevant cases.
